# Osteoporosis and associated risk factors in patients with severe hemophilia A: a case-control study from China

**DOI:** 10.1186/s12891-023-06795-y

**Published:** 2023-08-17

**Authors:** DongXiao Wu, ShaoNing Shen

**Affiliations:** 1 The First Clinical College of Zhejiang Chinese Medical University, The First Affiliated Hospital of Zhejiang Chinese Medical University, 548# BinWen Road, HangZhou, ZheJiang Province People’s Republic of China; 2 The Second Affiliated Hospital of Zhejiang Chinese Medicine University, 318 # Chaowang Road, HangZhou, ZheJiang Province People’s Republic of China

**Keywords:** Osteoporosis, Hemophilia, Risk factors

## Abstract

**Introduction:**

People with hemophilia risk osteoporosis more than healthy people, which may be related to specific factors.

**Methods:**

This case-control study included 53 patients with severe hemophilia type A and 49 healthy participants. Dual-energy X-ray absorptiometry (DXA) was used to determine bone mineral density (BMD). Collected information on age, body mass index (BMI), number of joint arthropathies, functional independence score in hemophilia (FISH), bone turnover markers, antibodies, treatment modalities. Identified independent risk factors for osteoporosis.

**Results:**

The BMD of the femoral neck (0.80 g/cm^2^vs.0.97 g/cm^2^), ward’s triangle (0.62 g/cm^2^vs.0.83 g/cm^2^), tuberosity (0.63 g/cm^2^vs.0.80 g/cm^2^) and hip (0.80 g/cm^2^vs.0.98 g/ cm^2^) in the case group were significantly lower than those in the control group, all of which were P < 0.001. However, there was no significant difference in the overall BMD of lumbar spine(L1-L4) (1.07 g / cm^2^vs. 1.11 g / cm^2^). The frequency of osteoporosis in the case group was 41.51%. BMI and FISH score were considered as independent risk factors for BMD decrease.

**Conclusion:**

The BMD of patients with severe hemophilia A is much lower than that of healthy population, and this difference is mainly reflected in the hip. The clear influencing factors were low BMI and functional independence decrease. Osteoclast was active while osteoblast activity was not enhanced synchronously, which may be the pathological mechanism of BMD decrease.

## Introduction

Hemophilia A is a rare congenital recessive X-linked disorder caused by lack or deficiency of clotting factor VIII (FVIII). The hallmark clinical characteristic is bleeding (spontaneous or after trauma) into joints [1]. Its severity is associated with.

FVIII activity, and severe hemophilia A is defined as FVIII activity of less than 1%.

Osteoporosis is a systemic bone disease characterized by osteopenia, increased bone fragility, and an increased risk for fractures. Osteoporosis in men is a growing concern, with clear risk factors including age, alcoholism, hypogonadism, etc. [2]. Hemophilia has not been defined as an obvious cause of secondary osteoporosis. However, several studies have shown that osteopenia is common among hemophiliacs [3], [4]. Thanks to the development of coagulation factor replacement therapy, hemophiliacs can now live almost as long and have a quality of life as the normal population. Identifying osteoporosis and associated risk factors in hemophiliacs is critical. At present, no studies have reported the frequency of osteoporosis in patients with severe hemophilia type A in China. To this end, we conducted this study to investigate osteoporosis in Chinese hemophiliacs and identify associated risk factors.

## Materials and methods

This case-control study included 53 patients with severe hemophilia A who visited the first affiliated hospital of Zhejiang university of traditional Chinese medicine as the case group and 49 healthy volunteers of the same sex and age as the control group. Exclusion criteria included: (1) continuously taking glucocorticoid drugs for more than 3 months; (2) hypogonadism; (3) thyroid and parathyroid disease; (4) retrovirals are in use; (5) alcoholism; (6) calcium, vitamin D, bisphosphonate and disumab are in use; 7.hip replacement; 8. presence of metal implants at the bone densitometry site. This study followed the declaration of Helsinki and was approved by the ethics committee of the first affiliated hospital of the Zhejiang university of Chinese medicine, with a unique ethics number: 2021-KL-104-01, and all participants signed the informed consent form.

### Bone densitometry

Dual-energy X-ray absorptiometry (DXA) is the gold standard for bone densitometry. Dual-energy X-ray bone densitometry (GE Lunar DPX Prodigy, YM0070331) was used to measure the total lumbar spine(L1-L4) and the left hip, including femoral neck, ward’s triangle, tuberosity, hip. The absolute value of bone mineral density (BMD) at each measurement site was expressed in g/ cm^2^. According to the World Health Organization’s (WHO) classification system [5], patients older than 50 years are recommended to use the T-score, with the T-score of < − 2.5 standard deviations defined as osteoporosis, the T-score between − 1 and − 2.5 standard deviations defined as osteopenia, and the T-score of >-1 standard deviation considered average compared with healthy young people of the same sex. The Z-score is recommended for patients under 50 years of age, and the score is compared to the expected BMD level of an age-matched healthy population. The Z-score of − 2 standard deviation or lower is considered “below age expectations,“ and the Z-score above − 1 standard deviation is considered normal [6].

### Demographic information

According to the questionnaire, the patient’s age (years), gender, height (m), weight (kg), and body mass index (BMI) (kg/m^2^) were calculated.

### Treatment modalities

Questionnaires were conducted to investigate the treatment modalities of hemophiliacs, including on-demand treatment and long-term regular prophylaxis. The age at start, duration, dose and frequency of administration of prophylactic treatment were recorded.

### Functional independence

Functional independence in hemophiliacs was assessed using the functional independence score in hemophilia (FISH) [7], which set independence for seven activities under three categories: self-care (grooming and eating, bathing, and dressing), transfer (chair and floor), and mobility (walking and going up and down stairs). Depending on the amount of help the patient needs to perform each function, it is divided into grades 1 to 4, which are scored as 1–4 points, respectively, and the total score of FISH is 7–28 points.

### Joint arthropathies

A Physical examination was performed on 10 joints of both hips, knees, ankles, shoulders, and elbows. Joint mobility less than the normal range are defined as joint arthropathies, and their number is recorded [8].

### Laboratory indicators

Blood tests are done to detect bone turnover markers and antibodies. Bone turnover markers include β-Cross Laps of type I collagen-containing cross-linked C-telopeptide(β-CTx), 25-hydroxyvitamin D (25(OH)D), calcium, phosphorus, parathyroid hormone (PTH), N-terminal peptide of type I procollagen (P1NP), osteocalcin (OC), and calcitonin. Antibodies include hepatitis B surface antigen (HBsAg), hepatitis C antibody (anti-HCV), and HIV antibody (anti-HIV).

### Statistical analysis

All data were analyzed descriptively using SPSS 23.0 (SPSS, Chicago, IL, USA) statistical analysis; the Shapiro-wilk test was performed on continuous variables, and continuous variables that conformed to the normal distribution were analyzed using the independent sample t-test or t’ test. Statistics were expressed as mean (standard deviation) for continuous variables that did not conform to the normal distribution. The Whimartney U-rank sum test was used for continuous variables that did not conform to the normal distribution, and the statistic was expressed as the median (minimum, maximum). Dicategorical and hierarchical variables were compared using either the Chi-square test or Fisher’s exact test, and the statistic was expressed as n. (%). Spearman’s rho correlation coefficient method was used to analyze correlations between continuous variables, while Kendall’s tau-b was used for correlation analysis between hierarchical variables. Multivariate linear regression was used for screening independent risk factors. The significance level is defined as P < 0.05.

## Results

### Participant characteristics

A total of 53 men with severe hemophilia type A (from 20 to 64 years old) and 49 healthy male volunteers of similar age (from 21 to 61 years old) were included in this study. Patients in the case group had the previous lowest FVIII activity of < 1%. There were no statistically significant differences in age and height between the two groups (P = 0.248, P = 0.323), while the difference in weight and BMI were statistically significant (P = 0.006, P = 0.001). The clinical characteristics, laboratory indicators and DXA results of participants in the two groups are shown in detail in Table 1.

**Table 1:** Clinical characteristics, laboratory parameters, and results of DXA scans in case and control groups

Parameters	Case group (n = 53) mean (SD)	Control group (n = 49) mean (SD)	*t/z/x2*	*P*
Age(years)	38.11(7.87)	36.41(6.86)	1.162	0.248
Height(m)	1.74(0.06)	1.73(0.08)	0.994	0.323
Weight(kg)	61.27(12.97)	67.79(10.48)	-2.780	0.006
BMI(kg/m^2^)	20.20(4.09)	22.72(3.10)	-3.521	0.001
β-CTx(ng/L)	777.15(253.78)	599.60(137.74)	4.435	<0.001
25(OH)D(nmol/L)^a^	68.96(31.92/117.92)	82.39(30.79/114.92)	− 0.854	0.393
Calcium(mmol/L)^a^	2.28(1.93/2.50)	2.33(2.17/2.54)	-1.394	0.163
Phosphorus(mmol/L)^a^	1.10(0.83/1.57)	1.23(0.84/1.54)	-1.193	0.233
PTH(pmol/L)^a^	4.05(1.60/7.49)	5.09(1.75/6.86)	-1.708	0.088
P1NP(ug/L)^a^	50.74(13.19/139.60)	52.64(11.49/88.12)	-1.082	0.279
BGP(ug/L)^a^	15.28(6.16/24.10)	18.18(6.03/24.5)	-1.279	0.201
Calcitonin(pmol/L)^a^	1.06(0.00/4.50)	1.19(0.03/2.79)	− 0.013	0.989
Average daily sunlight exposure(hours)^a^	1.5(0.5/3.0)	1.9(0.5/6.0)	− 0.342	0.732
FISH	14.26(4.52)			
HBV^b^	18(33.96%)	13(26.53%)		0.519
HCV^b^	12(22.64%)	0(0%)		<0.001
HIV^b^	3(5.66%)	0(0%)		0.244
**L1-L4**				
BMD(g/cm2)	1.07(0.121)	1.11(0.11)	-1.653	0.102
T-score	-0.10(0.99)	0.21(0.91)	-1.679	0.096
Z-score	0.08(0.98)	0.29(0.91)	-1.115	0.268
**Femoral neck**				
BMD(g/cm^2^)	0.80(0.13)	0.97(0.08)	-8.045	<0.001
T-score	-1.38(0.96)	-0.06(0.64)	-8.170	<0.001
Z-score	-1.15(0.96)	0.06(0.57)	-7.725	<0.001
**ward’s triangle**				
BMD(g/cm^2^)	0.62(0.14)	0.83(0.13)	-7.970	<0.001
T-score	-1.75(0.92)	-0.33(0.84)	-8.106	<0.001
Z-score	-1.41(0.97)	-0.12(0.70)	-7.726	<0.001
**Trochanter**				
BMD(g/cm^2^)	0.63(0.13)	0.80(0.08)	-8.197	<0.001
T-score	-1.60(1.04)	-0.23(0.67)	-7.964	<0.001
Z-score	-1.46(1.04)	-0.19(0.60)	-7.665	<0.001
**Hip**				
BMD(g/cm^2^)	0.80(0.14)	0.98(0.08)	-8.180	<0.001
T-score	-1.46(1.06)	-0.10(0.59)	-8.062	<0.001
Z-score	-1.40(1.05)	-0.09(0.55)	-7.986	<0.001

^a^Median (IQR)

^b^n(%)

### Bone mineral density

The BMD of the femoral neck (0.80 g/cm^2^vs.0.97 g/cm^2^), ward’s triangle (0.62 g/cm^2^vs.0.83 g/cm^2^), tuberosity (0.63 g/cm^2^vs. 0.80 g/cm^2^) and hip (0.80 g/cm^2^vs.0.98 g/cm^2^) in the case group was significantly lower than that in the control group, all of which were P < 0.001. However, there was no significant difference in overall BMD of lumbar spine (L1-L4) (1.07 g/cm^2^vs. 1.11 g/cm^2^), P = 0.102. According to the WHO classification system, patients under 50 years of age, assessed using the Z-scores, 19 of the 48 patients in the case group were " lower than expected for age,“ and 29 patients were considered normal. All 47 patients in the control group were normal, and none were below age expectations. The difference between the two groups was statistically significant, P < 0.001. Patients over 50 years of age were assessed using the T-score, among the 5 patients in the case group, 2 patients were classified as “osteopenia” and 3 patients as “osteoporosis”. Compared with 2 patients in the control group, 1 was defined as “normal” and 1 was “osteopenia.“ There was no significant difference between the two groups. P = 0.082, and the frequency of osteoporosis in the case group was 41.51%. Table 2 shows the classification of BMD by age.

**Table 2:** DXA scan results in terms of T- and Z‐scores, by age

DXA results	Case group(n = 53)		Control group(n = 49)	*P*	
**According to Z-score for patients < 50 y**					
Normal		29(54.72%)	47(95.92%)		< 0.001
Lower than expected for age		19(35.85%)	0(0.00%)		
**According to T-score for patients ≥ 50 y**					
Normal	0(0.00%)		1(2.04%)	0.082	
Osteopenia	2(3.77%)		1(2.04%)		
Osteoporosis	3(5.66%)		0(0.00%)		

### Bone turnover markers

The mean value of β-CTx in the case group was 777.15 (253.78) ng/L, while the mean value in the control group was 599.60 (137.74) ng/L, and the difference between the two groups was statistically significant (P < 0.001). The normal level of β-CTx in our laboratory was 43–783 ng/L, according to this standard, a total of 27 patients in the case group were outside the normal range, compared with only 4 mildly elevated in the control group. The median 25(OH)D in the case group was 82.39 nmol/L compared to 68.96 nmol/L in the control group. Vitamin D deficiency was defined as 25(OH)D < 30 nmol/L, and based on this criterion, no patient has vitamin D deficiency. The normal range for calcium was 2.1 to 2.6 mmol/L, with 2 patients in the case group (1.93mmol/L, 2.04mmol/L) were below the normal range, while all participants in the control group were within the normal range. The normal range for phosphorus was 0.81 to 1.65 mmol/L, with both groups of participants in the normal range. The normal range for PTH was 1.59 to 6.89 pmol/L, with 1 patient in the case group (7.49 pmol/L) exceeding the normal range, while all participants in the control group were within the normal range. The normal range of P1NP was 9.06–76.24 ug/L, with 10 patients in the case group outside the normal range (minimum 77.40 ug/L and maximum 139.60 ug/L), while 8 participants in the control group exceeded the normal range (minimum 78.00 ug/L, maximum 85.50 ug/L). The normal range for BGP was 6.02–4.66 ug/L, with both groups of participants in the normal range. The normal range of Calcitonin was 0-2.79 pmol/L, with 1 patient (4.5 pmol/L) in the case group being outside the normal range and all participants in the control group in the normal range. There were no significant differences in 25(OH)D, calcium, phosphorus, PTH, P1NP, BGP, and osteocalcin between the two groups (P = 0.393, P = 0.163, P = 0.233, P = 0.088, P = 0.279, P = 0.201, P = 0.989).

### History of seropositivity

There were 18 (33.96%) patients in the case group who were positive for HbsAg and 13 (26.53%) patients in the control group who were positive for HbsAg, and there was no significant difference between the two groups (P = 0.519). There were 12 (22.64%) HCV-positive patients in the case group, 3 (5.66%) patients were HIV-positive, and no participants in the control group were HCV or HIV antibody positive. The difference in anti-HCV positivity between the two groups was statistically significant (P < 0.001), while the difference in anti-HIV positivity was not statistically significant (P = 0.244).

### Joint arthropathies and FISH

Physical examination was performed in all patients to identify joint arthropathies, and none of the participants in the control group had joint arthropathies. In the case group, patients had at least 1 joint arthropathy and up to 7 joint arthropathies, with a median of 3. FISH scores were collected only in questionnaires conducted in the case group, with a minimum of 7 points and a maximum of 24 points. The case groups were divided into osteoporosis and regular. The differences between subgroups in joint arthropathies and FISH scores were further analyzed. The results are shown in Table 3.

### Treatment modalities

According to the statistics of coagulation factor replacement treatment in the case group, a total of 3 patients were treated on-demand, and 50 patients were treated with tertiary prophylaxis, that is, preventive treatment was started after the diagnosis of joint disease was clarified. No patients were treated with primary or secondary prophylaxis. The duration of preventive treatment ranged from 2 months to 60 months. Prophylactic therapeutic doses ranged from 5.5 IU/kg to 44.4 IU/kg. After dividing the case group into osteoporosis and regular. Further analyses were made for differences in duration and prophylactic therapeutic dose between subgroups. The results are shown in Table 3.

**Table 3:** Joint arthropathies, FISH and treatment modalities in case and control groups

Parameters	Normal group(n = 31)	Osteoporosis group(n = 22)	*P*
FISH^a^	16.00(4.53)	11.82(3.23)	.001
**Joint count** ^b^	2(1/6)	4(2/7)	<.001
**Hip**			
Normal	16(30.19%)	0(0.00%)	<.001
Unilateral	10(18.87%)	7(13.21%)	
Bilateral	5(9.43%)	15(28.30%)	
**Knee**			
Normal	2(3.77%)	0(0.00%)	<.001
Unilateral	22(41.51%)	4(7.55%)	
Bilateral	7(13.21%)	18(33.96%)	
**Ankle**			
Normal	20(37.74%)	11(20.75%)	.229
Unilateral	10(18.87%)	9(16.98%)	
Bilateral	1(1.89%)	2(3.77%)	
**Shoulder**			
Normal	26(49.06%)	18(33.96%)	.419
Unilateral	5(9.43%)	2(3.77%)	
Bilateral	0(0.00%)	2(3.77%)	
**Elbow**			
Normal	25(47.17%)	14(26.42%)	.118
Unilateral	6(11.32%)	7(13.21%)	
Bilateral	0(0.00%)	1(1.89%)	
**Treatment**			.563
On-demand Treatment	1(1.89%)	2(3.77%)	
Long-term regular tertiary prophylaxis	30(56.60%)	20(37.74%)	
Age at start(years)^a^	35.15(5.76)	36.42(9.61)	.585
Therapy time(month)^a^	29.33(18.29)	33.50(18.71)	.438
Prophylactic dose(IU/Kg)^a^	20.69(9.55)	25.77(10.09)	.078

^a^Mean(SD)

^b^Median (IQR)

### Correlation analysis of BMD

In the correlation analysis between BMD and other variables, BMI was positively correlated with BMD in each part. Positive β-CTx and anti-HCV were significantly negatively correlated with BMD in all sites. FISH scores were significantly positively correlated with BMD in the femoral neck, ward’s triangle, tuberosity, and hip. There was no significant correlation with lumbar spine(L1-L4) BMD. The number of joint arthropathies was significantly negatively correlated with BMD of the femoral neck, ward’s triangle, tuberosity, and hip. There was no significant correlation with lumbar spine(L1-L4) BMD. Other variables (age, 25(OH)D, calcium, phosphorus, PTH, P1NP, BGP, calcitonin, age at start and dose and duration of prophylaxis, HbsAg positivity, anti-HIV positivity) were not significantly correlated with bone mineral density at each site. The results are shown in Table 4.

**Table 4:** Correlation analysis between DXA measurements and other variables

		BMI	β-CTx	HCV	FISH	The number of joint arthropathies
L1-L4 BMD(g/cm^2^)	r	0.185	− 0.164	− 0.171	0.163	− 0.189
	P	0.006	0.015	0.037	0.094	0.063
Femoral neck BMD(g/cm^2^)	r	0.394	− 0.259	− 0.192	0.393	− 0.426
	P	<0.001	<0.001	0.019	<0.001	<0.001
ward’s triangle BMD(g/cm^2^)	r	0.441	− 0.258	− 0.230	0.442	− 0.501
	P	<0.001	<0.001	0.005	<0.001	<0.001
Trochanter BMD(g/cm^2^)	r	0.378	− 0.241	− 0.191	0.471	− 0.624
	P	<0.001	<0.001	0.019	<0.001	<0.001
Hip BMD(g/cm^2^)	r	0.466	− 0.278	− 0.190	0.547	− 0.635
	P	<0.001	<0.001	0.019	<0.001	<0.001

### Multivariate regression analysis

Multivariate linear regression analysis was performed using BMD as the dependent variable of each site, and no independent risk factors for the reduction of lumbar spine(L1-L4) BMD were found. BMI and FISH scores were independent risk factors for decreased BMD of the femoral neck, ward’s triangle, tuberosity, and hip.

## Discussion

Although many national and regional studies have reported the relationship between hemophilia and osteoporosis, there are no studies that report the current status of osteoporosis in hemophilia patients in China. The number of hemophilia patients in China is as high as 140,000 [9], and a trial in China is necessary to verify the relationship between hemophilia and osteoporosis. In this study, the BMD of the four sites of the femoral neck, ward’s triangle, tuberosity, and total hip of hemophilia patients was significantly lower than that of healthy controls, but the difference in the lumbar spine was not significant. This is similar to the conclusions of some previous studies [10–12]. The frequency of osteoporosis in our case group is as high as 41.51%, which may reflect the actual situation of hemophilia patients in China.

Age is a recognized risk factor for osteoporosis. However, there was no significant correlation between age and bone mineral density in our study, which may be associated with the high concentration of age of the participants we included. Therefore, the effect of age was not reflected in the statistical analysis. BMI has been an essential factor in BMD in both healthy people and people with other diseases [13, 14]. High BMI puts a more mechanical load on bones, increasing bone remodeling and thus increasing bone mass to bear greater loads. In our study, the BMI of the case group was significantly lower than that of the healthy control group. The low BMI puts a less mechanical load on hemophilia patients. This partly explains why load-bearing joints (e.g., hip joints) are more common in hemophiliac osteoporosis, while non-load-bearing joints (e.g., lumbar spine) are relatively normal.

We examined bone turnover markers and explored the relationship with BMD. β-CTx is one of the degradation products of collagen type I, which is present in the blood as an intact immunogenic protein, and collagen type I is the most abundant organic substance in the bone matrix. When physiologic or pathological bone resorption is enhanced, the degradation of type I collagen is also increased, and the corresponding decomposition fragment is increased in peripheral blood [15, 16], so the detection of β-CTx can reflect the degree of bone resorption. In our study, the β-CTx in the case group was significantly higher than in the control group (777.15 ng/L vs. 599.60 ng/L), indicating the degree of bone resorption in the case group was much higher than that in the control group. In the correlation analysis, β-CTx was significantly negatively correlated with BMD at all sites. This is similar to the conclusion of Katsarou [17] et al. Type I collagen is the most abundant collagen type in the human body, with an extended peptide chain at the amino (N-terminus) and carboxyl (C-terminus) procollagen. These extended peptide chains (properties) are cleaved by specific proteases during the conversion of procollagen to collagen. When mature collagen is formed, it is deposited in the bone matrix. The determination of PINP reflects the deposition of type I collagen so that PINP can be used as a marker of bone formation [18]. In our study, there was no significant difference in P1NP between the case and control groups, and there was no significant correlation between BMD and P1NP in the correlation analysis. This suggests that bone formation activity is similar in hemophilia patients to healthy patients. From this, we speculate that the pathological mechanism of osteoporosis in hemophilia patients may be that osteoclast activity is enhanced, and osteoblastic activity is not enhanced to the same extent. Several studies have reported that vitamin D deficiency is common in hemophiliacs and may be associated with osteoporosis and fragility fractures [11], [8], [19]. The main reason for vitamin D deficiency in hemophilia patients is restricted exposure to sunlight, which is associated with limited outdoor activities [20]. Based on this, we recommend that hemophilia patients living indoors increase the duration of sunlight exposure. All participants in this study were from southeast China, which received sufficient sunlight, and the average daily sunlight exposure in the case group was similar to that of the control group (1.5vs.1.9 h, P = 0.732). None of the participants used vitamin D supplements. Fortunately, none of the patients in our study were deficient in vitamin D. But in areas with limited sunlight, routine screening of hemophilia patients and the use of vitamin D supplements is critical. No correlation between 25(OH)D and BMD was found in the correlation analysis. In addition, there were no significant differences in PTH, BGP, and calcitonin between the case and control groups, and no significant correlation with BMD was found in the correlation analysis.

Osteoporosis is a known complication in patients with chronic liver disease [21]. The increased bone resorption through the receptor activator of nuclear factor kappa (RANK)-RANK ligand (RANKL)-osteoprotegerin (OPG) system and upregulation of inflammatory cytokines and decreased bone formation through increased bilirubin and sclerostin and lower insulin-like growth factor-1 are important mechanisms for osteoporosis in patients with liver disease [22]. We investigated the frequency of HBV and HCV in patients and analyzed the relationship with osteoporosis. The frequency of HBV was high in both the case group (18 cases, 33.96%) and the control group (13 cases, 26.53%). However, there was no significant correlation between HBV and BMD. Anti-HCV positivity was common in the case group (12 cases, 22.64%), while no patients in the control group were anti-HCV positivity. This is attributed to the increased risk of hematogenous infection due to the need for frequent blood transfusions in hemophiliacs. In the correlation analysis, HCV and BMD had a significant negative correlation. We only measured HbsAg and anti-HCV positive, not viral DNA content, so our conclusions only demonstrate a relationship between previous infection history and BMD.

Previous cross-sectional surveys have found that osteoporosis is common in AIDS patients [23]. There were 3 (5.66%) HIV-positive patients in the case group and no HIV-positive patients in the control group. There was no significant association between HIV positivity and BMD, which may be associated with fewer cases. Notably, the use of antivirals has an impact on BMD [24]. Overall, short-term BMD is lost by 1 to 2% over 2 to 4 years when antiviral therapy is initiated, followed by an increase or long-term stabilization of BMD [23]. None of our patients are on antiviral therapy, and it was found through questioning that none of the previously infected patients were receiving complete standard antiviral therapy. Therefore, we were unable to analyze the effects of antiviral therapy on osteoporosis.

Decreased physical activity is a known risk factor for osteoporosis, and we used the FISH score to assess functional independence and reflect the amount of daily activity in people with hemophilia. Patients in the case group had a FISH score distribution of 7 to 24 points, and their functional independence was significantly lower than that of healthy people. The FISH score was positively correlated with BMD of the femoral neck, ward’s triangle, tuberosity, and hip. Because of recurrent bleeding that begins in childhood, hemophiliacs often choose to avoid activities to reduce the occurrence of bleeding, which leads to a decrease in peak bone mass [25]. Joint arthropathies lead to decreased mobility, which in turn affects BMD. However, whether joint destruction itself affects BMD is unclear. Repeated intra-articular hemorrhage deposits hemosiderosis on the synovial surface, causing hypertrophic synovitis, and further damage to cartilage and subchondral bone [26]. BMD is most likely affected in this pathological process. Khawaja et al. [27]found that the degree of joint destruction was significantly negatively correlated with BMD at bilateral hips, femoral necks, and greater trochanters. Sossa [28] et al. came to similar conclusions. We counted the number of joint arthropathies per patient. We found that the number of joint arthropathies was significantly negatively correlated with BMD at the femoral neck, ward’s triangle, tuberosity, and hip. However, this effect was no longer significant in multivariate regression analysis. Therefore, it can be determined that joint arthropathies mainly affect BMD by reducing the amount of activity. More research is needed to explore the effects of joint pathological disruption on BMD.

Long-term regular prophylaxis has been shown to significantly protect BMD in children with hemophilia [27]. However, the role of long-term regular prophylaxis has not been demonstrated in adult hemophilia patients [29]. Some scholars have found that long-term deficiency of FVIII is an independent risk factor for osteoporosis and proposed that the mechanism may be FVIII:vWF complex inhibits osteoclast production and differentiation through the RANKL-OPG pathway [30]. Based on this theory, long-term regular prophylaxis should be beneficial for BMD. We investigated the treatment modalities of hemophiliacs, and all but 3 patients received on-demand treatment, and the remaining 50 patients received long-term regular tertiary prophylaxis. We counted the duration and dose of long-term regular prophylaxis. However, in the correlation analysis, no benefit of them was found for BMD. This contradicts previous conclusions, which may be that since our patients are on tertiary prophylaxis, Bone damage is well established before prophylactic therapy begins. Short-term preventive treatment cannot improve this bone destruction. More long-term follow-up studies are needed to assess the role of preventive treatment. We are well aware of the tremendous benefits of preventive treatment in improving bone density in hemophiliacs.

## Conclusion

This case-control study is from hemophiliacs in China. Patients with severe hemophilia type A have much lower BMD than healthy people, and this difference is mainly reflected in hips. Definite influencing factors are low BMI and reduced functional independence. Active osteoclasts and osteocyte activity without simultaneous enhancement may be pathological mechanisms of decreased BMD. Based on the current study, hemophiliacs are advised to ensure their nutritional intake and avoid low BMI. In addition, it is recommended that long-term regular prophylaxis should be carried out early. On the one hand, it can reduce the degree of joint destruction by bleeding and improve functional independence, on the other hand, appropriately increasing weight-bearing activities under the protection of coagulation factors can improve BMD.

## Data Availability

Data cannot be provided due to identifying information of participants but are available from the corresponding author on reasonable request.
